# Ultrasound for monitoring different stages of post-transplant lymphoproliferative disorder in a transplanted kidney: A case report and review of the literature

**DOI:** 10.1097/MD.0000000000036206

**Published:** 2024-02-23

**Authors:** Zu-Sheng Du, Xiao-Hong Xie, Jin-Jiao Hu, Ye Fang, Lu Ye

**Affiliations:** a Department of Ultrasound, Ningbo Yinzhou No.2 Hospital, Ningbo, China.

**Keywords:** contrast-enhanced ultrasound, post-transplant lymphoproliferative disorder, transplant kidney, ultrasound

## Abstract

**Rationale:**

Post-transplant lymphoproliferative disorder (PTLD) is a well-recognized, but uncommon complication in patients with kidney transplantation, which poses challenges in diagnosis and poor prognosis due to its low incidence and nonspecific clinical manifestations. As a routine follow-up examination method for kidney transplant patients, ultrasound (US) plays a significant role in the diagnosis of PTLD. Therefore, it is critical to evaluate the ultrasonic characteristics of PTLD in transplanted kidney patients for early detection and diagnosis.

**Patient concerns:**

A 59-year-old female patient was unexpectedly found with a mass in the hilum of the transplanted kidney 12th month after transplantation, which gradually grew up in the following 4 months. The latest US examination found hydronephrosis. Contrast-enhanced ultrasound (CEUS) demonstrated a hypo-enhancement pattern in arterial and parenchymal phases and showed a new irregular area lacking perceivable intensification within the mass, which was considered necrosis. Meanwhile, the patient developed an acute increase in serum creatinine from 122 to 195 μmol/L.

**Diagnosis:**

A US-guided biopsy was conducted with the final pathological diagnosis of PTLD (polymorphic).

**Interventions:**

After receiving 3 times of rituximab and symptomatic treatment, blood creatinine returned to normal but the mass was still progressing in the patient. Therefore, the treatment approach was modified to immune-chemotherapy.

**Outcomes:**

The patient was in a stable condition to date.

**Lessons:**

PTLD is a rare complication in a transplanted kidney. US and CEUS are the preferred imaging methods in renal transplant patients due to their good repeatability and no nephrotoxicity. This case demonstrates that continuous dynamic monitoring by using US and CEUS has significant value in the detection and diagnosis of PTLD in a transplanted kidney, suggesting early clinical intervention to avoid further progression.

## 1. Introduction

Post-transplant lymphoproliferative disorder (PTLD) is a rare but severe complication of solid organ transplantation, which is frequently related to the infection of Epstein-Barr virus (EBV) and cytomegalovirus (CMV).^[1]^ Only 1% of kidney transplant recipients will develop PTLD, which is substantially lower than other organ recipients.^[2,3]^ Early detection and diagnosis of PTLD in transplanted kidneys are crucial for prognosis, enabling patients to receive treatment as soon as possible.^[4]^ As the first-line imaging method for routine follow-up after kidney transplantation, ultrasound (US) has the advantages of convenience, non-invasiveness, and non-radiation, which has become an essential tool for discovering and differentiating substantive causes in transplanted kidneys.^[5]^ With the development of US technology, the application of contrast-enhanced ultrasound (CEUS) in kidney transplantation is increasingly mature and extensive, which can display real-time microcirculation blood perfusion in transplanted kidney tissue with unique advantages such as good repeatability and no nephrotoxicity. CEUS provides a good prospect for early detection and diagnosis of postoperative complications and improvement of patient survival rate after kidney transplantation.^[6,7]^

In this case, a 59-year-old female was diagnosed with PTLD in a renal allograft after various examinations. This is the third report on CEUS manifestations of PTLD in a transplanted kidney. We described the US and CEUS features of the PTLD in different stages. Furthermore, we summarized the ultrasonic characteristics by conducting a comprehensive literature review.

## 2. Case presentation

**Figure 1.**
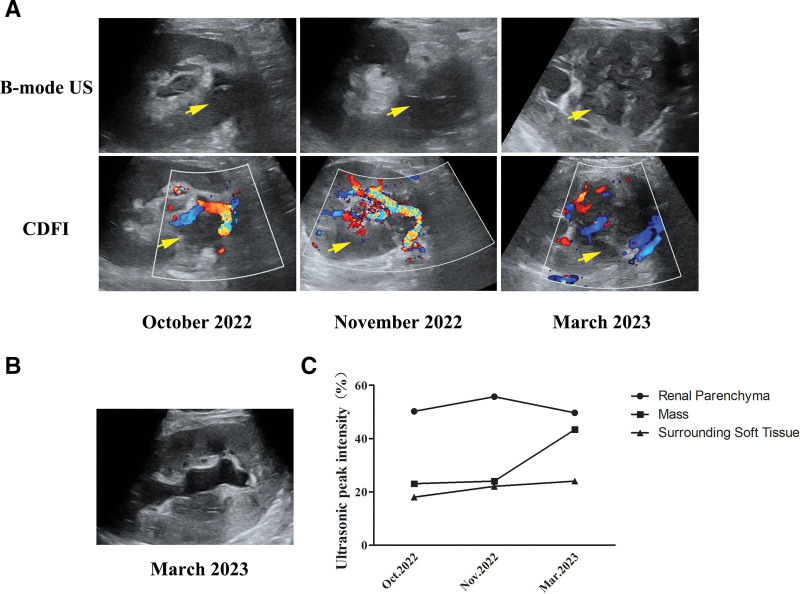
B-mode ultrasound (US) and Color Doppler flow imaging (CDFI) of the patient. (A) B-mode US and CDFI images demonstrated a poorly defined, hypovascular mass (arrows) in the hilum of the transplanted kidney. The size of the mass gradually enlarged, and the internal echo gradually decreased from isoechoic to heterogeneous (hypoechoic mainly) during the 3 examinations. (B) Hydronephrosis in the transplanted kidney. (C) The contrast peak intensity of the mass, renal parenchyma, and surrounding soft tissue separately. The contrast peak intensity in the mass was lower than that in the parenchyma and it was markedly increased in the examination of March 2023. No significant difference was found between the mass and the soft tissue in the first 2 examinations. CDFI = Color Doppler flow imaging.

**Figure 2.**
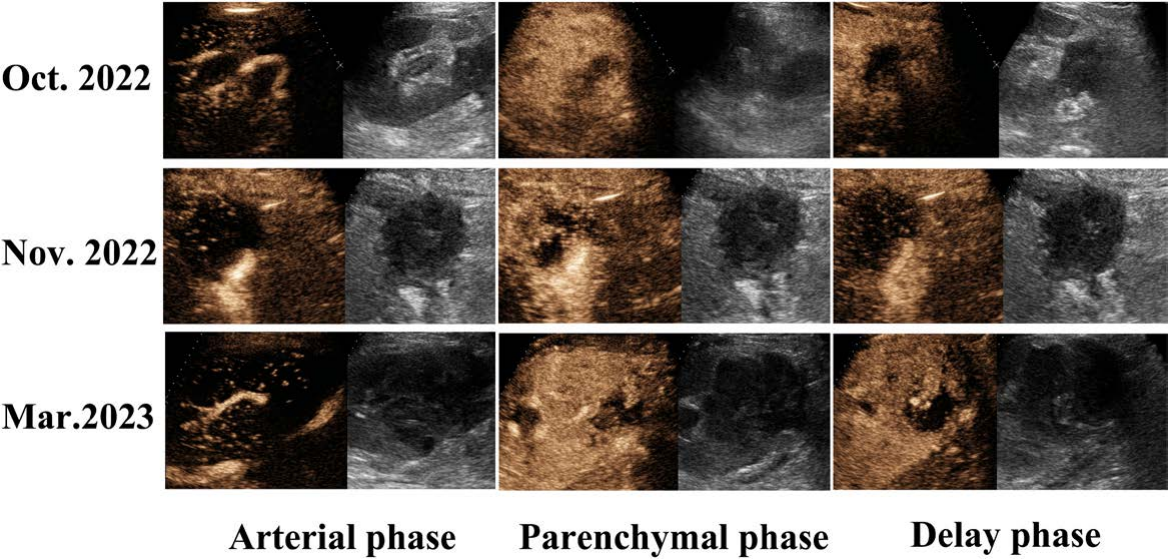
Contrast-enhanced ultrasound (CEUS) of the patient. The CEUS examinations in October and November 2022 showed the mass exhibited a centripetal hypo-enhancement pattern. The examination in March 2023 showed an increase in contrast intensity of the mass but still manifested the hypo-enhancement pattern, and a new irregular area lacking perceivable intensification within the mass.

This patient is a 59-year-old female who had a kidney transplant 16 months ago. Prior to being diagnosed with PTLD, she has been hospitalized twice. The mass in the renal hilum of a transplanted kidney was accidentally found in her first hospitalization due to deep vein thrombosis of the right lower limb (12 months after kidney transplantation). The US revealed an ill-defined isoechoic-hypoechoic mass located in the renal hilum (5.0 × 4.0 cm) of the transplanted kidney, which showed irregular morphology and unclear boundary with surrounding soft tissue. Color Doppler flow imaging (CDFI) showed no blood flow signal inside the mass. Meanwhile, we found that the mass was adjacent to the renal artery, and no obvious abnormal stenosis or increased flow velocity was detected (Fig. 1A). Due to the difficulty of differentiating the boundary between ill-defined mass and transplanted kidney by conventional ultrasonography, after injecting the contrast agent, the edge of the mass started enhancing at 15 seconds, which manifested as a gradual centripetal enhancement, demonstrating a hypo-enhancement pattern. The mass achieved peak enhancement at 38 seconds, the dynamic CEUS images indicated that the intensity was lower than that in the surrounding kidney parenchyma (Fig. 2). Then, the quantitative analysis demonstrated that the contrast peak intensity of the mass was equivalent to the surrounding soft tissue, while was lower than that in the transplanted renal parenchyma in the first 2 examinations (Fig. 1C).

Half a month later, the patient was re-hospitalized due to repeated fever. The US showed that the mass was a little larger than before, with a size of approximately 5.2 × 3.7 cm and the internal echo was lower. The edge of the mass was still unclear and was indistinguishable from the surrounding soft tissue. No blood flow signal was found within the mass (Fig. 1A). CEUS revealed no significant changes in the enhancement pattern of the mass (Fig. 2). The serum creatinine was normal, and the patient denied any specific symptoms during the regular follow-up; hence, she refused further diagnosis and treatment.

However, the patient was hospitalized for the third time in March 2023 due to recurrent fever. The US showed mild hydronephrosis and the mass was further enlarged (7.4 × 6.4 cm), with markedly heterogeneous internal echogenicity. CDFI demonstrated some rod-shaped blood flow signals within the mass (Fig. 1A–B). CEUS showed that the edge of the mass started enhancing at 13 seconds, and achieved peak enhancement at 23 seconds, which was equivalent to the surrounding kidney parenchyma (Fig. 2). Besides, CEUS showed an extra irregular unenhanced region within the mass, which was considered necrosis (Fig. 2). Compared with the previous CEUS examination, the boundary between the mass and the renal parenchyma was more unclear. Furthermore, the quantitative analysis revealed that the contrast peak intensity of the mass was higher than that in the first 2 examinations, lower than that in the transplanted renal parenchyma, and higher than that in the surrounding soft tissue (Fig. 1C).

Meanwhile, laboratory tests indicated that the patient developed a sharp increase in serum creatinine from 122 to 195 μmol/L over a few days. Besides, the blood examination presented both CMV-IgG antibody positivity and EBV DNA positivity.

Due to the significant enlargement of the mass, the presence of hydronephrosis, necrotic areas, and elevated blood creatinine levels, further consideration has been given to the mass, and malignant tumors cannot be ruled out.

**Figure 3.**
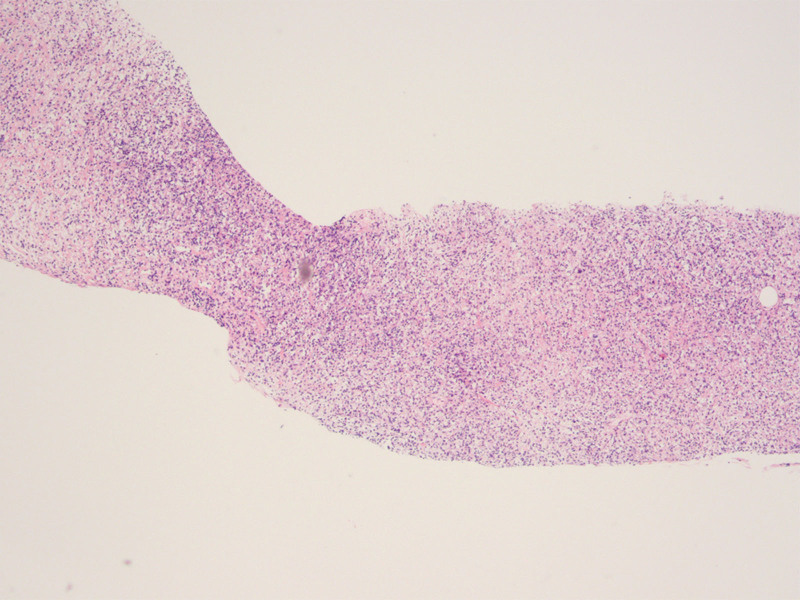
Pathological findings of US-guided biopsy. Hematoxylin and eosin staining revealed diffuse infiltration of atypical lymphoid cells. (40 ×). US = ultrasound.

**Figure 4.**
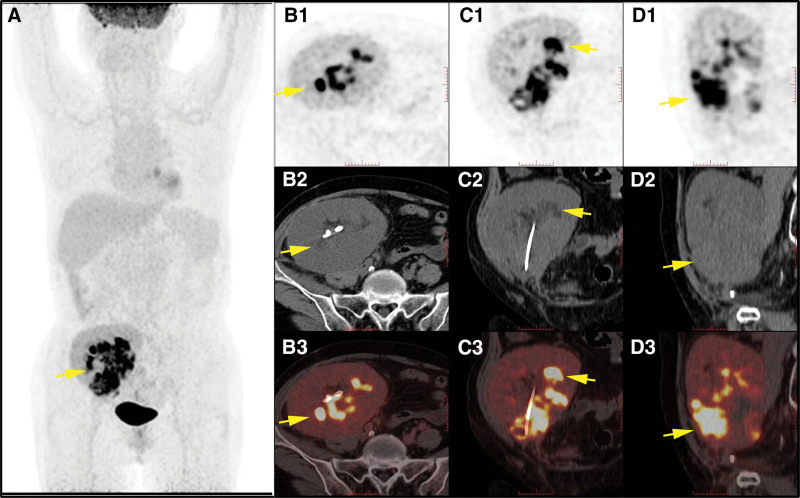
^18^F-FDG PET/CT of the patient after 3 times of rituximab treatment. CT scan showed the transplanted kidney was enlarged and swollen, and the cortex was thickened. Multiple poorly circumscribed and irregular low-density shadows were observed in the renal allografts. (A) ^18^F-FDG PET MIP image shows a quantity of intense uptake in the right lower quadrant (SUVmax = 15.7). The B1, C1, and D1 (PET), B2, C2, and D2 (CT), and B3, C3, and D3 (fused PET/CT) showed multiple abnormal FDG-avid lesions in the transplanted kidney (arrows) in axial, sagittal and coronal sections separately. No other significant abnormal FDG-avid lesion is observed in the rest PET study of the entire body (including the brain).

Subsequently, a biopsy was conducted guided by the US, leading to the final pathological diagnosis of PTLD (polymorphic), with immunohistochemistry staining showing positive results for CD20 (B-cell marker) and CD3 (T-cell marker) (Fig. 3). The patient received 3 times of rituximab and symptomatic treatment, and blood creatinine was returned to normal but the mass still progressed. Positron emission tomography-computed tomography revealed extra multiple lesions in the transplanted kidney (Fig. 4). Transplantation doctors modified the treatment approach to immune chemotherapy and the patient was in a stable condition to date.

Ethical approval was not necessary, as the article is a case report, which is based on the clinical information of the patient. Informed consent was obtained from the patient for the purpose of publication.

## 3. Discussion

PTLD was first considered a complication of kidney transplantation as early as 1968,^[8]^ which is rare, challenging, and associated with a high mortality rate.^[9]^ EBV infection is one of the most common risk factors for PTLD, with over 2-thirds of PTLD patients reported to be EBV-positive. In addition, CMV and hepatitis virus infections can also increase the risk of PTLD. Other risk factors including the types of allograft transplantation, treatment for acute rejection, immunosuppression, and patient age were also linked to PTLD occurrence.^[9–11]^ According to National Comprehensive Cancer Network guidelines, PTLD is classified into nondestructive lesions, monomorphic PTLD (B-cell type), polymorphic PTLD, monomorphic PTLD (T-cell type) and classical Hodgkin lymphoma PTLD, and the treatment options for PTLD vary based on these types and may include rituximab therapy or chemo-immunotherapy.^[12]^

PTLD in a transplanted kidney may present various clinical symptoms as follows: fever, increased serum creatinine, dysuria, pain, hypertension, and painless gross hematuria (Table 1). However, the above manifestations are not specific enough to remind transplant doctors to associate them with PTLD. Therefore, as the preferred imaging examination for daily follow-up of kidney transplant recipients, the US plays a crucial role in the early detection and diagnosis of PTLD.^[23,29]^ To improve the understanding and sensitivity of the ultrasonic features of PTLD in a transplanted kidney making it possible to diagnose it in a reliable way.

**Table 1:** Cases of PTLD in transplanted and native kidney with ultrasonic findings.

Ref.	Age/Sex	Time post-transplant (Mos.)	Initial symptom	Concomitant infection		Ultrasonic findings	Outcome	Pathologic findings
				CMV	EBV			
^[13]^	67/M	120	/	-	/	(1).Centrally septated, relatively echo-free mass.	Died	Polymorphic diffuse B-cell lymphoma
^[14]^	24/M	9	(1).Fever; (2).Increased serum creatinine; (3).No lymphadenopathy or hepatomegaly by physical examination.	/	/	(1).The renal pelvis was involved by a 7.5 × 8.0 cm ill-defined isoechoic mass and extending into the renal parenchyma; (2).Mild hydronephrosis.	Alive well	Malignant lymphoma
^[15]^	55/M	5	(1).Increased serum creatinine;	/	/	(1).Hydronephrosis.	Alive well	Monoclonal B cell Lymphoproli- feration
^[16]^	14/M	4.5	(1).Fever; (2).Dysuria; (3).Left abdominal and flank pain; (4).A history of trauma.	/	/	(1).A large, poorly circumscribed, heterogeneous echoic mass impinged upon the allograft pelvis; (2).Hydronephrosis.	/	A high-grade malignant lymphoma (small noncleaved cell, non-Burkitt subtype)
^[17]^	45/M	3	(1).Increased serum creatinine.	/	+	(1).9.5 × 8.5 cm fluid collection anteromedial to a hydronephrotic transplanted kidney.	Alive well	Polymorphic diffuse B-cell lymphoma
^[18]^	52/M	8	(1).Increased serum creatinine; (2).Poorly controlled hypertension (170/110 mm/Hg); (3).No fever.	/	-	(1).Hypoechoic mass (5 × 5 cm) in the hilum of the renal allograft extended along the renal vessels; (2).Moderate hydronephrosis; (3).More hypoechoic and irregular mass (rehospitalized in 2 mo later).	Alive well	Polymorphic diffuse B-cell lymphoma
^[19]^	44/F	6	(1).Suddenly increased blood pressure (180/100 mm Hg) and weight (2 kg); (2).Moderately increased serum creatinine (172 µmol/L); (3).Fever, cough, dyspnea and hypoxemia.	/	+	(1).A tight stenosis (approximately 85%) with very high peak systolic velocities (3.5 m/s); (2).A 4 cm hypoechoic mass was detected around the artery at the level of the stenosis; (3).No hydronephrosis.	Alive well	Monoclonal B cell Lymphoproliferation
^[20]^	18/M	6	/	/	+	(1).Ureteral obstruction caused by a renal altograft-based mass (4 × 6 × 6 cm).	Alive well	Monoclonal B cell Lymphoproliferation
^[21]^	52/M	8	(1).Painless gross hematuria; (2).Normal serum creatinine (1.9 mg/dL)	+	/	(1).Hydronephrosis in the allograft kidney; (2).A 6 cm mass involved the lower posteromedial aspect of the graft.	Died	Monoclonal B cell Lymphoproliferation
^[22]^	59/M	30	(1).14 days of mandibular sensory abnormalities; (2).Renal failure caused by long-term use of cyclosporin A therapy; (3).Fever.	+	/	(1).A 7 cm globular mass pressed into the renal transplant hilum.	Died	Polymorphic diffuse B-cell lymphoma
^[23]^	54/M	144	(1).Weight loss; (2).Severe low backache; (3).Pain in the right iliac fossa radiating to the right leg.	/	/	(1).A thick-walled cystic mass behind the lower pole of the transplant kidney; (2).Mass was flanked by the external and internal iliac vessels, adherent to the lesion.	Alive well	Polymorphic diffuse B-cell lymphoma
^[24]^	37/M	6	(1).Macrohematuria.	/	+	(1).A low echoic area in the renal graft.	Alive well	Diffuse large B-cell lymphoma
^[25]^	30/M	17	(1).Routine follow-up.	/	/	(1).A mass (5.5 × 4.3 cm) located between the liver and the atrophic right kidney; (2).The mass showed markedly heterogeneous internal echogenicity; (3).No blood signal within the mass.	Alive well	Diffuse large B-cell lymphoma
^[26]^	77/M	108	(1).A hypertensive crisis.	/	+	(1).Well-defined, discrete, hypoechoic, solitary, renal mass (2.1 × 3.3 × 2.8 cm); (2).Minimal blood signal within the mass; (3).CEUS: Enhancing, but hypovascular both in arterial and parenchymal phases.	Alive well	Diffuse large B-cell lymphoma
^[27]^	52/M	156	(1).Fever for the past 4 mo; (2).A weight loss of 3 kg over the last 3 mo; (3).Normal serum creatinine (1.2 mg/dL)	+	-	(1).A hypoechoic space occupying lesion (1.5 × 1.4 cm) at lower pole of the right native kidney.	Died	Monomorphic T-cell PTLD
^[1]^	54/F	2.5	/	/	+	(1).A clearly hypoechoic solid mass at the renal hilum (1.6 × 1.9 × 1.4 cm); (2).2 clearly hypoechoic solid masses measuring 1.7 × 1.5 × 1.7 and 2.1 × 1.9 × 1.5 cm (6 mo later); (3).the masses were not invading the peripheral vessels, but expanding extrinsically in the renal sinus and pelvis; (4).CEUS: heterogeneous echogenicity, peripheral enhancement with central nonenhancement.	Alive well	Malignant pleomorphic lymphoma
^[28]^	69/F	11	(1).Progressive myasthenia both lower limbs; (2).Weight loss; (3).Normal creatinine and urea.	+	+	(1).No abnormalities in the transplanted kidney; (2).Multiple cystic lesions in atrophic untransplanted kidneys; (3).CEUS: a completely homogeneously enhancement mass (1.3 × 1.1 cm) in the medial portion of the left untransplanted kidney.	Died	Diffuse large B-cell lymphoma

CEUS = contrast-enhanced ultrasound, CMV = cytomegalovirus, EBV = Epstein-Barr virus, PTLD = post-transplant lymphoproliferative disorder.

We collected 15 cases of PTLD in a transplanted kidney with US descriptions and summarized them in Table 1. The location and size of the mass were variable, which may relate to the patient clinical manifestations and the development of PTLD. The mass was generally located at the hilum of the transplanted kidney with a large size when it was first detected, with a poorly circumscribed border and irregular morphology. The internal echo of PTLD was more specific compared to other features. Most masses presented with heterogeneous echotexture, which was mainly hypoechoic, and some masses were iso-echoic. An anechoic area could be found in the later stage, indicating necrosis and the progression of the PTLD. In addition, Mohammad et al reported a case of PTLD which was presented as a painful lymphocele. The US showed a thick-walled cystic mass behind the lower pole of the transplanted kidney and pathologic findings revealed that the cyst wall was necrotic lymphoid tissue.^[23]^ Furthermore, ureteral obstruction caused by the mass could be easily observed by the US.^[18]^ When the patient suddenly exhibited increased blood pressure, it might be associated with the stenosis of the allograft artery caused by the mass, and the US could make a prompt effective evaluation of the degree of stenosis.^[19]^

In our case, the ill-defined mass in the hilum of the transplanted kidney grew larger over time and the internal echo gradually changed from isoechoic to hypoechoic, until the internal anechoic area was observed in the later stage of the PTLD. CDFI showed that there was no blood flow signal within most of the masses, which was consistent with the early manifestations of the mass in our case. However, blood flow signals could be detected within the mass in the later stage. The above results indicated that the development of PTLD is a dynamic process, which exhibited the US as a valuable follow-up observation method for the different stages of PTLD.

Compared to contrast enhanced-magnetic resonance imaging and contrast enhanced-computed tomography, real-time microcirculation blood perfusion makes CEUS a remarkable technology in monitor the lesions in a transplanted kidney, which is no nephrotoxicity and would be more suitable for patients with renal dysfunction. CEUS can quantitatively display the blood flow perfusion of the graft by selecting and measuring different regions of interest of the transplanted kidney, with the advantage of identifying transplant complications.^[30,31]^

This is the third case of CEUS manifestations of PTLD in a renal transplant. The first one by Alyssa Lampe et al in 2015 suggested the hypo-enhancement in the transplant renal mass both in arterial and parenchymal phases, and there was no enhancement in the center of the mass, which was considered as necrosis.^[26]^ Li et al reported the second one in 2018. They found that CEUS showed striking heterogeneous echogenicity in the mass. Specifically, early peripheral enhancement with central non-enhancement was recorded and it was suggested necrosis.^[1]^

In this case, there are similarities in the enhancement pattern of CEUS compared to the aforementioned cases. In the early stages of the PTLD (US examinations in October and November 2022), the mass exhibited a centripetal hypo-enhancement pattern. With the development of the PTLD, hydronephrosis and new irregular non-enhancement area appeared (US examinations in March 2023), and the latter was considered necrosis. The boundary between the mass and the renal parenchyma was more unclear as compared to the former examination. Moreover, we further quantitatively analyzed the contrast peak intensity of the mass, parenchyma, and surrounding soft tissue separately. The results revealed that the contrast peak intensity in the mass was lower than that in the parenchyma, and there was no significant difference between the mass and the soft tissue in the initial stage (Fig. 1C), while an increase in the contrast peak intensity was detected after the mass progression. Thus, quantitative results further demonstrated the superiority of CEUS in the dynamic monitoring and evaluation of blood perfusion in PTLD. Finally, the US-guided biopsy was conducted and the pathology confirmed the diagnosis of polymorphic PTLD. Notably, US-guided biopsy is already a mature technology and has been widely used in clinical practice. However, when encountering a mass with unclear boundaries between adjacent tissues, it has a certain possibility of failure. Here, CEUS can effectively compensate for this by displaying the boundaries in real-time, distinguishing the parenchyma and the solid portion and necrotic areas within the mass, which further improves the success rate of puncture and diagnosis.^[28,32,33]^

In addition to the occurrence of PTLD in the transplanted kidney, we also found 2 cases of PTLD in the native kidney after renal transplant with US records,^[27]^ which indicated that while paying attention to transplanted kidneys, the native kidneys should also be monitored on a daily basis. Furthermore, when PTLD occurs in the central nervous system, gastrointestinal tract, lung, larynx, and cauda equina,^[21,34,35]^ the role of the US is limited, and it should be combined with other imaging examinations such as computed tomographu, magnetic resonance imaging, and Positron emission tomography-computed tomography. Besides, the patient clinical manifestations and previous laboratory findings are important to provide critical information for diagnosis, and doctors can determine whether further examination is necessary.

The study also suffers from limitations. Obviously, the low incidence of PTLD in transplanted kidneys limits the association of this disease with space-occupying lesions detected by doctors. In this case, a lack of understanding of the ultrasonic features of PTLD in the transplanted kidney and no specific symptoms in the patient led to the delayed diagnosis. In addition, although CEUS is particularly important in kidney transplant patients due to the lack of renal toxicity, there are also some variables involved in the implementation of this technology, such as the injection volume of contrast agents and analysis of microvascular flow, which may affect the detailed diagnosis of diseases.

In summary, the US as the preferred imaging method in renal transplant patients is crucial for the early diagnosis of PTLD. With the application of CEUS, the ultrasonic characteristics of PTLD in a transplanted kidney are more distinctive. If the renal transplant patient has a fever, elevated serum creatinine, hydronephrosis, and atypical US findings (exhibiting heterogeneity over time), especially when CEUS indicates hypo-enhancement pattern and necrosis within the mass, the sonographer, and clinician should be cautioned to consider PTLD within the differential diagnosis.

## Author contributions

**Conceptualization:** Zu-Sheng Du, Xiao-Hong Xie.

**Data curation:** Zu-Sheng Du.

**Investigation:** Zu-Sheng Du, Xiao-Hong Xie, Jin-Jiao Hu, Lu Ye.

**Methodology:** Zu-Sheng Du, Xiao-Hong Xie, Jin-Jiao Hu, Ye Fang.

**Project administration:** Ye Fang, Lu Ye.

**Visualization:** Jin-Jiao Hu, Ye Fang, Lu Ye.

**Writing – original draft:** Zu-Sheng Du, Xiao-Hong Xie, Jin-Jiao Hu, Ye Fang, Lu Ye.

**Writing – review & editing:** Zu-Sheng Du, Xiao-Hong Xie, Ye Fang, Lu Ye.

## References

[1] Li J Liu Y Wang Z. Multimodality imaging features, treatment, and prognosis of post-transplant lymphoproliferative disorder in renal allografts: a case report and literature review. Medicine (Baltim). 2018;97:e0531. 10.1097/MD.0000000000010531 PMC5944490 29703027

[2] Yaginuma T Yamamoto H Mitome J. Successful treatment of monomorphic primary central nervous system post-transplantation lymphoproliferative disorder 5 years after kidney transplantation. Transpl Infect Dis. 2012;14:E102–106. 22931101 10.1111/j.1399-3062.2012.00781.x

[3] Olsen SR Bhutani M. Multiple cavitating nodules in a renal transplant recipient. Can Respir J. 2009;16:195–7. 20011727 10.1155/2009/521548 PMC2807794

[4] Opelz G Dohler B. Lymphomas after solid organ transplantation: a collaborative transplant study report. Am J Transplant. 2004;4:222–30. 14974943 10.1046/j.1600-6143.2003.00325.x

[5] Li Marzi V Campi R Sessa F. Standardized duplex ultrasound-based protocol for early diagnosis of transplant renal artery stenosis: results of a single-institution retrospective cohort study. Biomed Res Int. 2018;2018:2580181. 29850493 10.1155/2018/2580181 PMC5925009

[6] Goyal A Hemachandran N Kumar A. Evaluation of the graft kidney in the early postoperative period: performance of contrast-enhanced ultrasound and additional ultrasound parameters. J Ultrasound Med. 2021;40:1771–83. 33184930 10.1002/jum.15557

[7] Mueller-Peltzer K Negrao de Figueiredo G Fischereder M. Contrast-enhanced ultrasound (CEUS) as a new technique to characterize suspected renal transplant malignancies in renal transplant patients in comparison to standard imaging modalities. Clin Hemorheol Microcirc. 2018;69:69–75. 29630539 10.3233/CH-189114

[8] Doak PB Montgomerie JZ North JD. Reticulum cell sarcoma after renal homotransplantation and azathioprine and prednisone therapy. Br Med J. 1968;4:746–8. 4881420 10.1136/bmj.4.5633.746 PMC1912885

[9] Khedmat H Taheri S. Very late onset lymphoproliferative disorders occurring over 10 years post-renal transplantation: PTLD.Int. Survey. Hematol Oncol Stem Cell Ther. 2011;4:73–80. 21727768 10.5144/1658-3876.2011.73

[10] Claudon M Kessler M Champigneulle J. Lymphoproliferative disorders after renal transplantation: role of medical imaging. Eur Radiol. 1998;8:1686–93. 9866789 10.1007/s003300050614

[11] Lewis S Harber M Fernando B. Rapid and early diagnosis of posttransplant lymphoproliferative disease using cross-sectional nuclear medicine imaging and laparoscopic lymph node biopsy: a report of 2 cases. Exp Clin Transplant. 2017;15:470–3. 26744227 10.6002/ect.2015.0012

[12] Darmanis S Sloan SA Croote D. Single-cell RNA-seq analysis of infiltrating neoplastic cells at the migrating front of human glioblastoma. Cell Rep. 2017;21:1399–410. 29091775 10.1016/j.celrep.2017.10.030 PMC5810554

[13] Tubman DE Frick MP Hanto DW. Lymphoma after organ transplantation: radiologic manifestations in the central nervous system, thorax, and abdomen. Radiology. 1983;149:625–31. 6359259 10.1148/radiology.149.3.6359259

[14] Russ PD Way DE Pretorius DH. Posttransplant lymphoma. Sonographic characteristics of renal allograft involvement. J Ultrasound Med. 1987;6:453–6. 3305981 10.7863/jum.1987.6.8.453

[15] Gibel LJ Harford A Yogel LR. Renal transplant obstruction by B cell lymphoproliferative disorder: case report and review of the literature. J Urol. 1988;140:593–5. 2842516 10.1016/s0022-5347(17)41729-0

[16] Olcott EW Goldstein RB Salvatierra O. Lymphoma presenting as allograft hematoma in a renal transplant recipient. J Ultrasound Med. 1990;9:239–41. 2184248 10.7863/jum.1990.9.4.239

[17] Squiers EC West JC Leonard D. Epstein-Barr virus--associated posttransplant lymphoproliferative disorder presenting as perirenal transplant lymphocele. Transplantation. 1993;56:1278–9. 8249138

[18] Palmer BF Sagalowsky AI McQuitty DA. Lymphoproliferative disease presenting as obstructive uropathy after renal transplantation. J Urol. 1995;153:392–4. 7815594 10.1097/00005392-199502000-00030

[19] Hestin D Claudon M Champigneulles J. Epstein-Barr-virus-associated post-transplant B-cell lymphoma presenting as allograft artery stenosis. Nephrol Dial Transplant. 1996;11:1164–7. 8671992

[20] Kirn D Venook A Pena I. Pulmonary aspergillosis masquerading as progressive post-transplant lymphoma. Transpl Int. 1996;9:517–9. 8875798 10.1007/BF00336833

[21] Goral S Felgar R Shappell S. Posttransplantation lymphoproliferative disorder in a renal allograft recipient. Am J Kidney Dis. 1997;30:301–7. 9261047 10.1016/s0272-6386(97)90071-8

[22] Vavrinova H Viklicky O Lacha J. [Post-transplantation lymphoproliferation in patients with intensive immunosuppression]. Vnitr Lek. 2006;52:645–8. 16871771

[23] Khan MS Ahmed S Challacombe B. Post-transplant lymphoproliferative disorder (PTLD) presenting as painful lymphocele 12 years after a cadaveric renal transplant. Int Urol Nephrol. 2008;40:547–50. 18369734 10.1007/s11255-008-9367-2

[24] Miyagi S Sekiguchi S Kawagishi N. Rituximab therapy and reduction of immunosuppression to rescue graft function after renal posttransplantation lymphoproliferative disorder found by macrohematuria in a pancreas and kidney transplant recipient: a case report. Transplant Proc. 2011;43:3299–301. 22099782 10.1016/j.transproceed.2011.10.002

[25] Lee M Kim SK Chung YE. Necrotic lymphoma in a patient with post-transplantation lymphoproliferative disorder: ultrasonography and CT findings with pathologic correlation. Ultrasonography. 2015;34:148–52. 25541069 10.14366/usg.14050 PMC4372711

[26] Lampe A Duddalwar VA Djaladat H. Contrast-enhanced ultrasound findings of post-transplant lymphoproliferative disorder in a transplanted kidney: a case report and literature review. J Radiol Case Rep. 2015;9:26–34. 10.3941/jrcr.v9i10.2602 PMC4638398 26629291

[27] Chandra A Kaul A Aggarwal V. Native kidney posttransplant lymphoproliferative disorder in a renal transplant recipient. Saudi J Kidney Dis Transpl. 2017;28:942–4. 28748904

[28] Zhang JC Lan HX Zhao HJ. Application of contrast-enhanced ultrasonography in the diagnosis of post-kidney transplant lymphoproliferative disorder in native kidney- a case report. BMC Cancer. 2019;19:1135. 31752767 10.1186/s12885-019-6355-0 PMC6873515

[29] Kew CE 2nd Lopez-Ben R Smith JK. Postransplant lymphoproliferative disorder localized near the allograft in renal transplantation. Transplantation. 2000;69:809–14. 10755531 10.1097/00007890-200003150-00023

[30] Kihm LP Hinkel UP Michael K. Contrast enhanced sonography shows superior microvascular renal allograft perfusion in patients switched from cyclosporine A to everolimus. Transplantation. 2009;88:261–5. 19623023 10.1097/TP.0b013e3181ac8cdd

[31] Grzelak P Szymczyk K Strzelczyk J. Perfusion of kidney graft pyramids and cortex in contrast-enhanced ultrasonography in the determination of the cause of delayed graft function. Ann Transplant. 2011;16:48–53. 21436774

[32] Fan P Cao J Jin Y. Efficacy of contrast-enhanced ultrasound-guided percutaneous core needle biopsy in anterior mediastinal masses. J Interv Med. 2022;5:159–65. 36317148 10.1016/j.jimed.2022.04.006 PMC9617154

[33] Nolsoe CP Nolsoe AB Klubien J. Use of ultrasound contrast agents in relation to percutaneous interventional procedures: a systematic review and pictorial essay. J Ultrasound Med. 2018;37:1305–24. 29230842 10.1002/jum.14498

[34] El-Natout T Natout MA Hamdan AL. Post-transplant Lymphoproliferative Disease (PTLD) of the larynx in an adult with kidney transplant; a case report. J Voice. 2021;35:810.e7–8. 10.1016/j.jvoice.2020.01.014 32151514

[35] Nishiyama Y Iwanami A Mikami S. Post-transplant lymphoproliferative disorder of the cauda equina in a kidney transplant recipient. Spinal Cord Ser Cases. 2018;4:100. 10.1038/s41394-018-0135-y PMC6219478 30416756

